# Introduction to a community dataset from an infrasound array experiment at Mt. Etna, Italy

**DOI:** 10.1038/s41597-021-01030-6

**Published:** 2021-09-23

**Authors:** S. De Angelis, L. Zuccarello, S. Rapisarda, V. Minio

**Affiliations:** 1 School of Environmental Sciences, University of Liverpool, 4 Brownlow Street, L69 3GP Liverpool, UK; 2 Istituto Nazionale di Geofisica e Vulcanologia, Sezione di Pisa, via Cesare Battisti, 53, 56125 Pisa, Italy; 3 Istituto Nazionale di Geofisica e Vulcanologia, Sezione di Catania - Osservatorio Etneo, Piazza Roma, 2, 95125 Catania, Italy; 4 Dipartimento di Scienze Biologiche, Geologiche e Ambientali, Università Degli Studi di Catania, Piazza Università, 2, 95131 Catania, Italy

**Keywords:** Natural hazards, Solid Earth sciences

## Abstract

Volcanic activity represents a hazard to population and infrastructure worldwide. The study of acoustic waves in the atmosphere by volcanic activity is growing in popularity as an effective tool to monitor and understand the mechanisms of eruptions. In 2019, we deployed two 6-element infrasound arrays at Mt. Etna, Italy, one of the most active volcanoes in the world. Our experiment captured a range of acoustic signals associated with diverse activity ranging from background degassing to energetic Strombolian explosions, lava flows, and atmospheric injection of volcanic ash. Here, we present a description of this valuable, publicly available, research dataset. We document the design and scope of the experiment, report on data availability, and present a brief summary of the activity observed at Mt. Etna during our deployment aiming to facilitate future use of these valuable data. This dataset is the first example of open data from a multiple infrasound array experiment at Mt. Etna and one of the few available globally.

## Abstract (summary)

**Table:** 

Measurement(s)	acoustic waves • volcanic activity
Technology Type(s)	infrasound
Sample Characteristic - Environment	volcano
Sample Characteristic - Location	Mount Etna

Machine-accessible metadata file describing the reported data: 10.6084/m9.figshare.15144996

## Background & Summary

Volcanic activity is a prolific source of infrasound, i.e., acoustic atmospheric waves with frequencies below 20 Hz^1–3^. Continuous surface degassing, explosions and surface mass flows are common sources of infrasound at volcanoes. Infrasound-based methods are increasingly popular for real-time monitoring of volcanic unrest with applications at scales from local to global, that is source-receiver distances from few hundred meters to several thousand kilometres^3–7^. Infrasound sensors are often deployed – at the local scale – as distributed networks, within distances of up to several kilometres from one or multiple active vents. Alternatively, microphones can be installed as tight clusters of multiple sensors, or arrays, at distances of several tens of meters from one another^8^. Array deployments offer advantages over distributed networks owing to their performances in noise reduction and signal dis crimination. The wealth of infrasound data gathered at active volcanoes over the past two decades has allowed significant advances in our understanding of the processes that control the onset, temporal evolution, style and intensity of eruptions, and associated hazards^1,2^. Infrasound recorded at local distances from volcanic sources has been key to inform the development of methods to detect and track the evolution of eruptions in real-time, to compile and validate models that account for the influence of atmospheric conditions and topography on the propagation of the acoustic wavefield, and to unravel the links between acoustic sources and eruption intensity^9–13^. Despite a rapidly growing number of volcano infrasound studies, many questions remain open due to the comparatively scarce number of publicly available high-quality infrasound datasets^8^.

In the summer of 2019, we deployed two small-aperture infrasound arrays at Mt. Etna, Italy, within the framework of EUROVOLC (European Network of Observatories and Research Infrastructures for Volcanology), a project coordinated by the Icelandic Meteorological Office and funded under the Horizon 2020 program of the European Commission. One of the overarching goals of EUROVOLC is to improve integration within the volcanological community bringing together scientists from a wide range of disciplines, data and infrastructure. EUROVOLC provides competitive access to key infrastructure at a number of field locations across Europe. Within this framework we were granted access and logistics support for an infrasound experiment at Mt. Etna (VOSSIA, VOlcano monitoring with SeiSmic and Infrasound Arrays) through one of the EUROVOLC partner organizations, the Istituto Nazionale di Geofisica e Vulcanologia, Sezione di Catania-Osservatorio Etneo (INGV-OE). The core objectives of VOSSIA were:

1. test the potential of using multiple infrasound array to discriminate multiple active vents (e.g., ref. ^8^).
2. develop a fast and efficient real-time workflow for the analysis of infrasound data, including estimates of uncertainties on acoustic source locations (e.g., ref. ^8^).
3. investigate the potential integration of infrasound methods into volcano monitoring and early warning protocols at Mt. Etna.

The experiment recorded diverse infrasound linked to activity ranging from background degassing to eruption at one of the active craters at Mt. Etna. In this manuscript we introduce the dataset including details on the instruments and their deployment, and statistics on data recovery. We also provide a summary of the most notable activity observed at Mt. Etna during the recording period, showing examples of infrasound associated with such activity in order to facilitate future use of the dataset by other investigators.

### Mt. Etna, Italy

**Fig. 1:**
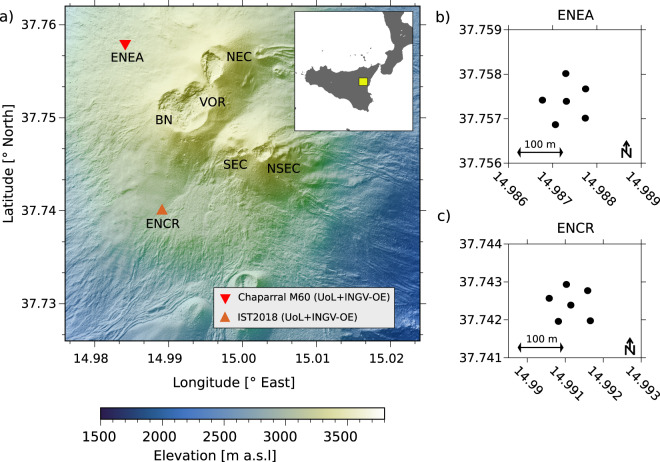
(**a**) Map of array sites ENEA and ENCR in the summit area of Mt. Etna. The active summit craters are marked (NEC, North East Crater; VOR, Voragine; BN, Bocca Nuova; SEC, South East Crater; NSEC, New South East Crater). The inset map shows the location of the Mt. Etna National park (yellow square) on the island of Sicily, in Southern Italy. (**b**) and (**c**) Configuration of the two infrasound arrays; each dot represents the location of one microphone.

Mt. Etna is located in Southern Italy (34.748° N, 14.999° E, summit elevation 3320 m) near the city of Catania, the second largest city on the island of Sicily (Fig. 1a,inset). Mt. Etna is a stratovolcano with predominately basaltic composition and activity ranging from lava effusion to Plinian eruptions^14,15^. Volcanic hazards at Mt. Etna are mainly linked to its location within 100 km of three airports^6^, its proximity to densely populated areas, and the elevated number of tourists visiting the Mt. Etna National Park every year.

Eruptions at Mt. Etna – over the past two decades – have been typified by lava effusion anticipated or punctuated by Strombolian explosions and intense lava fountain activity from one of the summit craters (Fig. 1): the North East Crater (NEC), Bocca Nuova (BN), Voragine (VOR), South East Crater (SEC) and New South East Crater (NSEC). In recent times, explosive activity at Mt. Etna has intensified with 38 eruptive episodes between 2011 and 2013^16^ and more sporadically with >30 episodes during 2014-present. Typical activity consists of sequences of Strombolian explosions transitioning to sustained lava fountaining – occasionally feeding large ash plumes – followed by the opening of one or more lateral vents at one the summit craters, and emplacement of lava flows. In the volcanological literature, these episodes of rapidly escalating and intensifying activity are referred to as paroxysms. Detailed information and catalogues of eruptive activity at Mt. Etna since 1995 can be found in refs. ^6,16^.

Since 2001, Mt. Etna has been monitored by INGV-OE with a multi-parameter instrument network, including continuous and real-time seismic, acoustic, deformation, gas, visual and thermal infrared measurements. As part of their routine monitoring protocols, INGV-OE produces real-time deformation, gas flux and seismic tremor amplitude time series, as well as catalogues of explosions and seismic tremor locations. Since 2001, INGV-OE has operated an eruption alert system based on empirical relations between the amplitude of seismic tremor and the occurrence of paroxysmal activity^17^. A second alert system, based on unsupervised neural network classification of seismic tremor, was later developed^18^ and is also currently used at INGV-OE. In addition to the INGV-OE network, the Laboratorio di Geofisica Sperimentale of the University of Firenze (LGS) also operates two four-element, real-time, infrasound arrays at Mt. Etna. The acoustic data collected forms the basis for an Early Warning System that has been in operation since 2015. Early Warning messages from this system are dispatched to the Italian Civil Protection headquarters and used to inform further action by the national and regional civil protection authorities^6^.

Past infrasound studies at Mt. Etna have been based on both data recorded by the 8-station INGV-OE infrasound network^19^, and occasional small-scale temporary experiments^20^. This body of research includes studies on: i) the characterization and location of acoustic sources^19^; ii) investigation of their source mechanisms^20–23^; iii) models of the relationships between acoustic signals and variable eruptive regimes^20^; iv) the links between the acoustic wavefield and eruption source parameters^11,22^.

## Methods and Technical Validation

Two 6-element infrasound arrays were deployed at Mt. Etna between 2 July and 26 August, 2019 by a team of researchers from the University of Liverpool (UK) and the INGV-OE within the framework of the VOSSIA experiment. Figure 1 shows the two array sites (ENEA and ENCR, Fig. 1a), and the locations of all acoustic sensors deployed (Fig. 1b,c). The arrays were deployed in similar configurations, consisting of a central element surrounded by 5 sensors positioned approximately at the vertices of a pentagon, with an aperture of approximately 100 meters (Fig. 1b,c). The locations of the two arrays were chosen considering three main criteria: i) site accessibility and safety of personnel; ii) minimizing differences in elevation between sensors within each array; iii) optimizing the detection and discrimination of activity from all summit craters. Table 1 provides information on locations for the sensors at the ENEA and ENCR arrays.

**Table 1:** Coordinates of infrasound sensors installed at the ENEA and ENCR array sites. Station Coordinates ^(*)^.

Array Name	Station Name	Latitude [°N]	Longitude [°E]	Elevation [m]
ENEA^(1)^	ENEA1	37.75789	14.98790	3043
	ENEA2	37.75710	14.98791	3071
	ENEA3	37.75685	14.98705	3060
	ENEA4	37.75743	14.98724	3053
	ENEA5	37.75823	14.98724	3039
	ENEA6	37.75734	14.98651	3038
ENCR^(2)^	ENCR1	37.74287	14.99170	3000
	ENCR2	37.74243	14.99117	2988
	ENCR3	37.74305	14.99098	2999
	ENCR4	37.74210	14.99188	2984
	ENCR5	37.74197	14.99089	2978
	ENCR6	37.74240	14.99029	2990

^(*)^ Coordinates are an average of ten individual GPS readings at each site.

^(1)^ Sensor type at this site is Chaparral Physics M60 UHP (see main text for additional details).

^(2)^ Sensor type at this site is IST2018 (see main text for additional details).

**Fig. 2:**
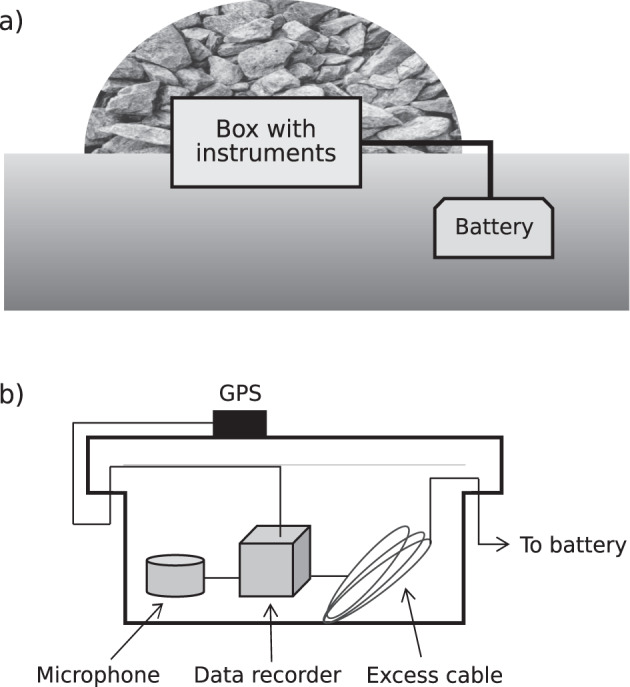
(**a**) Sketch of infrasound station design for the VOSSIA experiment. A plastic box containing all instrumentation was covered with rocks to provide drainage of rainwater and shielding from wind noise. The battery was buried at approximately 1–2 m distance from the instrument box; (**b**) Sketch of the internal layout of the instrument box.

All array elements at the ENEA site were equipped with Chaparral Physics M60 UHP microphones (http://chaparralphysics.com/model60.html, last accessed 19 March, 2021); the M60 UHP sensors have a sensitivity of 9 mV/Pa, flat frequency response (to within +/−3 dB) between 0.3 and 245 Hz, and full-scale pressure range of +/−1000 Pa. The microphones at the ENCR site were IST2018 with sensitivity of 20 mV/Pa, flat frequency response between 0.06 and 40 Hz, and a full-scale pressure range of +/−240 Pa. A comparison between the performances of these two types of sensors can be found in ref. ^24^. Data were sampled at 100 Hz using DIGOS DATACUBE^3^ digital data recorders (https://digos.eu/CUBE/DATA-CUBE-Datasheet-2017-02.pdf, last accessed 19 March, 2021) on their gain 1 setting (4,096 V peak-to-peak). DATACUBE recorders have an effective resolution of 22.4 bit (at 100 Hz), analog to digital conversion dynamic range of 125 dB (at 100 Hz), and a GPS timing accuracy of 1 ms. Voltage dividers (by a factor of 4.7) designed at the University of Liverpool were used in order to match the voltage output of the sensors to the input of the data recorders. All sensors were tested (24-hour ‘huddle’ test) before and after deployment. All stations were deployed in the same configuration; sensors, cables and data recorders were placed inside plastic boxes connected to 12V-75Ah batteries, which were buried at approximately 1–2 m from the recording equipment (Fig. 2). The boxes had a non-sealed cover to ensure effective coupling between of the microphone with the atmosphere. The GPS antenna was positioned outside the instrument box. Each box was then covered with rocks sourced on site to provide drainage of rainwater and shielding from wind noise. No additional noise reducing strategies were adopted.

## Data Records

The complete dataset described in this manuscript, including continuous raw waveform data and station metadata, is available through the facilities of the Incorporated Research

**Fig. 3:**
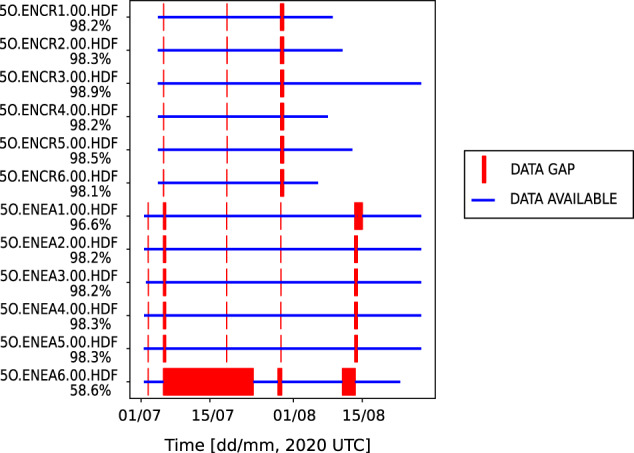
Summary of data availability for the ENEA and ENCR array.

Institutions for Seismology Data Management Center^25^. The data are archived under the temporary FDSN (International Federation of Digital Seismograph Networks) network code 5O (IRIS: MDA: 5O). Data availability statistics for the experiment are shown in Fig. 3. Data recovery was between 96.6% and 98.9% at all stations with the exception of the ENEA6 site (58.6%), which was affected by damage to the data recorder between 6 and 23 July, 2019. Other minor data gaps visible in Fig. 3 correspond to periods of equipment maintenance.

## Activity at Mt. Etna in July-August, 2019

In this section we provide a brief overview of the diverse activity at Mt. Etna during the period of our experiment (2 Jul – 26 Aug, 2019). We offer a preliminary account of the main eruptive events and their timing in order to facilitate future use of the dataset by other investigators. A detailed report, or an in-depth analysis of the mechanisms of eruptive activity, is beyond the scope of this manuscript. The activity reported by INGV-OE, and observed in the field by the experiment team, ranged from passive background degassing from the summit craters to two episodes of paroxysmal activity at the NSEC on 18–19 July and 27–28 July, 2019. A large explosion from the NSEC generated an ash plume rising to > 3 km above the vent on 27 July, 2019. Paroxysms followed a pattern that is typical at Mt. Etna; they started with rapidly intensifying Strombolian explosions, eventually feeding sustained lava fountains, finally followed by the emplacement of lava flows. Activity at the NSEC remained elevated throughout the entire month of July. The NEC was mainly active during the first half of July; degassing levels were elevated, and two large ash-rich explosions were observed on 2 and 3 July, 2019. For the remaining of the experiment activity at the NEC remained low, characterized by low-level degassing, occasionally punctuated by small gas-and-ash explosions. BN was also mostly active during the first half of July when sequences of intra-crater gas explosions were observed. A summary of all observations during the deployment period is provided in Table 2.

**Table 2:** Summary of activity at Mt. Etna (Italy) during the period 01/07/2019–18/08/2019.

TIME PERIOD	BN	NEC	NSEC
01/07–07/07	Deep intra-crater gas explosions.	Intense degassing (04/07–05/07). Two larger ash-rich explosions on 02/07 at 10:06 UTC and 03/07 at 10:11 UTC.	Intense degassing during 01/07–05/07 July. Discrete ash-rich explosions on 05/07 Strombolian activity on 06/07.
08/07–14/07	Deep intra-crater gas explosions.	Intense degassing all week. Two larger explosions on 08/07 at 20:45 UTC and 13/07 at 12:00 UTC.	Intense degassing all week. Strombolian explosions starting on 14/07 at 13:00 UTC from the southeasternmost vent.
15/07–21/07	Sporadic ash explosions on 19/07 and 21/07.	Sporadic ash explosions on 19/07 and 20/07.	Small ash explosions between 15/07 and 17/07. Vigorous Strombolian activity on 18/07 between 14:00 and 22:00 UTC. Strombolian activity on 19/07 between 12:00 and 20:00 UTC, and on 20/07 between 07:00 and 14:00 UTC. Lava flow activity starting on 18/07 at 23:09 UTC on the NE flank of the NSEC.
22/07–28/07	No activity observed.	Sporadic, minor, ash explosions on 28/07.	Irregular Strombolian explosions resume on 25/07. A new vent appears at 08:15 UTC on the southern flank of NSEC. At 9:20 UTC activity intensifies, including ash-rich explosions. Major ash-rich explosion on 27/07 at 12:21 UTC (ash plume > 3 km above the vent). Activity decreases on 28/07 starting at 03:40 UTC. Lava flow active between 27/07 at 08:15 UTC and 28/07 22:00 UTC on the south flank of the NSEC.
29/07–04/08	One ash explosion on 31/07 at 04:18 UTC.	Frequent, small, gas-and-ash explosions in the morning on 31/07.	No activity observed.
05/08–11/08	No activity observed.	Continuous background degassing with occasional minor emissions of ash. Partial collapse of NEC rim.	No activity observed.
12/08–18/08	No activity observed.	Continuous background degassing with occasional minor emissions of ash.	No activity observed.
19/08–25/08	Small explosions with minor ash content.	Continuous background degassing with occasional minor emissions of ash.	No activity observed.

## Examples of infrasound signals in the dataset

The diverse infrasound recorded during July-August 2019 holds potential to contribute to improving our understanding of eruption mechanisms at Mt. Etna, and to validate models of the generation and propagation of acoustic wavefields in volcanic areas (e.g., ref. ^11^). In addition, these data could be further exploited to test the performance and limitations of infrasound array processing workflows, with the objective to inform and optimize future deployments of acoustic sensors for volcano monitoring. The dataset has already been used to demonstrate that two optimally positioned arrays allow continuous detection and discrimination of activity from all summit craters at Mt. Etna, including estimates of uncertainty in these measurements^8^.

**Fig. 4:**
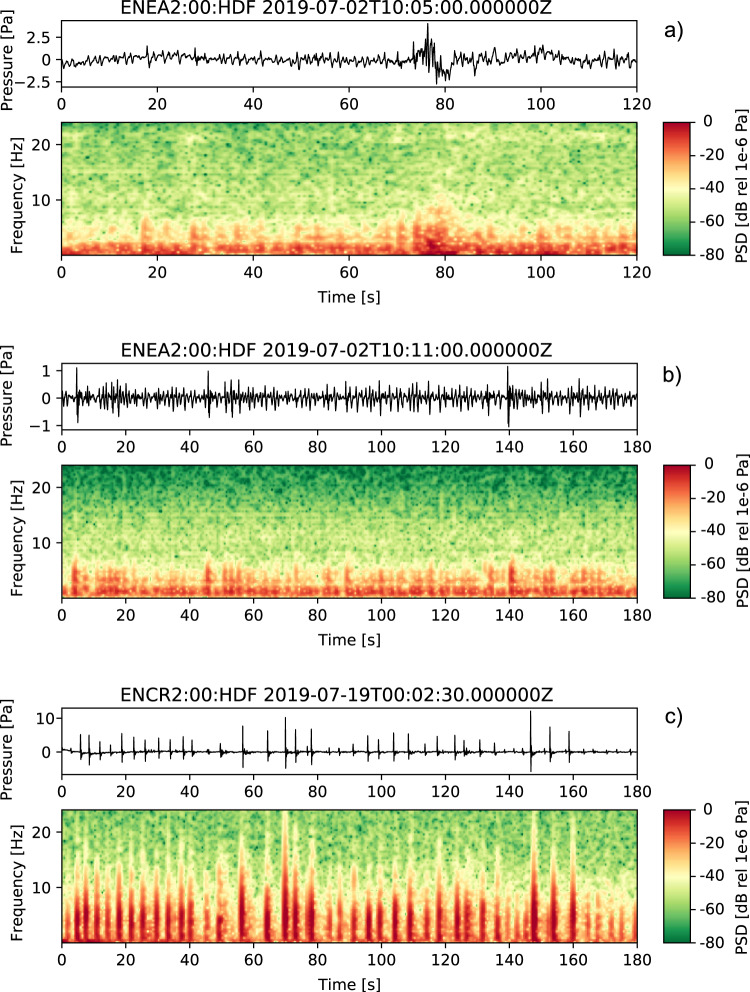
Infrasound waveforms recorded at Mt. Etna during July-August, 2019 and their spectrograms. Data are high-pass filtered (0.01 Hz) to remove the effect of long-period noise. (**a**) Ash explosion (~75–85 s) at the NEC; (**b**) Deep intra-crater gas explosions from BN; (**c**) Sequence of Strombolian explosions at the NSEC.

**Fig. 5:**
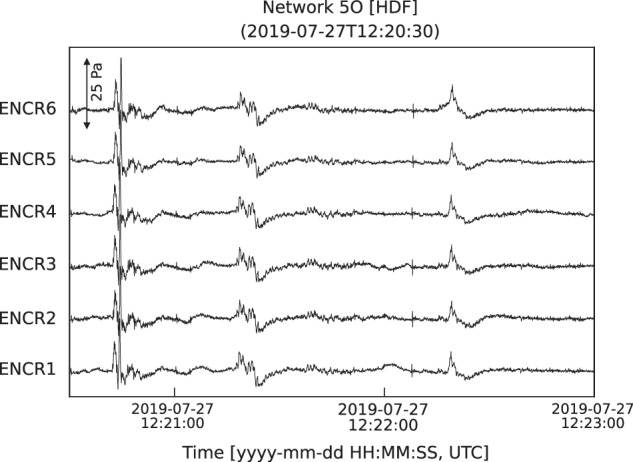
Infrasound waveforms recorded across the ENCR array on 27 July, 2019 during a major ash producing event. The onset of the even corresponds to the first large impulse, and the activity continued punctuated by lower-amplitude pulses.

**Fig. 6:**
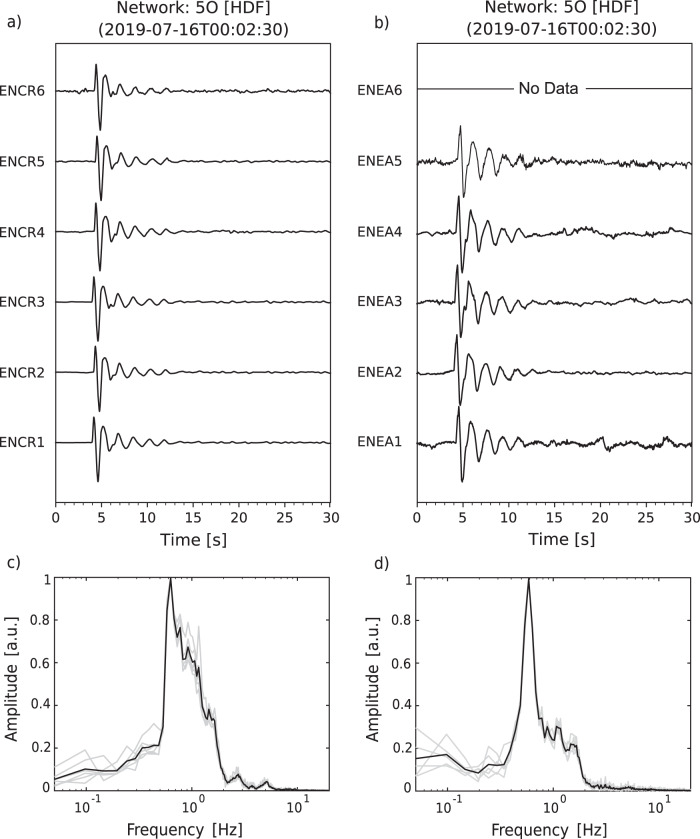
(**a**) and (**b**) Monochromatic waveforms recorded across the ENCR and ENEA arrays, respectively. Signals associated with intra-crater gas explosions within the BN crater (see main manuscript); (**c**) and (**d**) Individual Fourier frequency-amplitude spectra (light grey lines) and corresponding average spectra (black lines) for the waveforms shown in panels (**a**) and (**b**), respectively.

Here, we show examples of waveforms recorded during July-August 2019 associated with confirmed sources and, briefly, discuss their characteristics. The activity observed during our field experiment included episodic ash emissions, degassing from all summit craters, and Strombolian explosions. Infrasound signals corresponding to these types of activity, and their spectrograms, are shown in Fig. 4. The waveforms recorded during minor emissions of ash (e.g., Fig. 4a seconds 75–85) – typically observed at the NEC – exhibit low amplitudes (on the order of few Pa), emergent character (i.e., lack of an impulsive onset) and consist of multiple pulses. Their energy is concentrated in the 0.5–5 Hz frequency band (Fig. 4a), not dissimilar from gas explosions shown in Fig. 4b. Only one major ash explosion was observed during the deployment on 27 July, 2019 at approximately 12:21 UTC. The waveforms for this event recorded by all microphones at the ENCR array are shown in Fig. 5. Amplitudes are notably higher for this explosion than for the episodic ash emissions observed from the NEC (Fig. 5a). The signal has an impulsive onset at ~12:20:40 UTC, followed by at least two additional pulses; a sustained ash plume, rising to > 3 km above the vent, was reported by INGV-OE (Table 2). Sequences of impulsive, rapidly occurring and gas-rich, explosions were typical throughout the deployment, originating either from a vent located deep within the BN crater (Fig. 4b) or the NEC. Their infrasound signature consists of sequences of approximately N-shaped, low-amplitude, pulses at rates of up to one every second, often merging into one another to form a tremor-like signal (Fig. 4b). Elevated Strombolian activity was observed at the NSEC during the second half of July, accompanied by a lava flow originating on the NE flank of the NSEC (Table 2). Figure 4c shows 3 min of infrasound, including multiple Strombolian explosions. The signals corresponding to individual explosions are impulsive, characterized by a well-defined main pulse at times followed by one or more smaller trailing compressions. The spectrogram for these events (Fig. 4c) shows energy in the band from about 0.6 to 10 Hz, for the larger events reaching up to 20 Hz. Two additional characteristics of these explosion signals are the overall waveform amplitude modulation effect over time, and a degree of waveform asymmetry with the main compression phase having larger amplitude than the following decompression (Fig. 4c). Mt. Etna, similarly to other open-vent volcanoes where explosive degassing takes place (e.g., Johnson *et al*., 2018), produces waveforms with long ringing codas, characterized by strongly peaked, monochromatic, spectra. Figure 6 shows an example of such monochromatic signals as recorded across the ENEA and ENCR arrays along with their frequency spectra, showing a dominant peak at about 0.6 Hz. Visual observations conducted by the field team during the experiment confirmed that these signals were generated by intra-crater gas explosions either within the NEC or BN. The location of the explosion is also confirmed by previous results in ref. ^8^ crossing the back-azimuth obtained from array processing of ENEA and ENCR data. The source mechanisms of monochromatic signals at Mt. Etna have recently been investigated, for example in refs. ^21,22^, suggesting that the occurrence of strongly peaked spectra is linked to acoustic resonance at the crater, and thus, controlled by vent and crater morphology. The dataset presented in this manuscript includes numerous examples of such waveforms, thus providing a unique record to allow further investigation of the resonance mechanisms associated with intra-crater explosive degassing at Mt. Etna, and other volcanoes.

## Usage Notes

Data can be obtained in a number of standard seismological data file formats from IRIS DMC using one of their multiple data request tools (IRIS: Data at IRIS). Data can also be retrieved from the IRIS DMC data servers using open source software applications for processing of seismological time series such as the ObsPy package^26^.

## Data Availability

A Python notebook is provided in the supplementary material section of this manuscript, which demonstrates data retrieval from IRIS DMC. The notebook also demonstrates how to process data to obtain the results shown in Fig. 4. The user will require a working installation of the ObsPy package^26^ to execute the notebook^27^.
